# Investigating Sex-Linked miRNAs for Potential Osteoarthritis Therapy Biomarkers

**DOI:** 10.3390/ijms27021019

**Published:** 2026-01-20

**Authors:** Viviana Costa, Giulia Sacchi, Luca Andriolo, Giuseppe Filardo, Gianluca Giavaresi, Francesca Veronesi

**Affiliations:** 1 Surgical Sciences and Technologies, IRCCS Istituto Ortopedico Rizzoli, 40136 Bologna, Italy; giulia.sacchi@ior.it (G.S.); gianluca.giavaresi@ior.it (G.G.); francesca.veronesi@ior.it (F.V.); 2 Clinica Ortopedica e Traumatologica II, IRCCS Istituto Ortopedico Rizzoli, 40136 Bologna, Italy; luca.andriolo@ior.it; 3 Faculty of Biomedical Sciences, Università della Svizzera Italiana, 6900 Lugano, Switzerland; giuseppe.filardo@usi.ch

**Keywords:** miRNAs, osteoarthritis, biomarkers

## Abstract

Sex-specific factors can influence the onset and progression of osteoarthritis (OA), yet the molecular mechanisms underlying their impact remain poorly defined. This study investigated whether plasma microRNAs (miRNAs) correlate to sex-dependent OA progression, based on evidence of enhanced spontaneous osteoclastogenesis in peripheral blood mononuclear cells (PBMCs) derived from OA patients. miRNAs were evaluated on OA-plasma (n = 20 men, 20 women with knee OA; KL grade I–II) and their role on OA signaling was investigated through bioinformatic analysis. Seven miRNAs were identified as significantly upregulated in men’ vs. women’ samples: hsa-miR-107, hsa-miR-23a-3p, hsa-miR-103a-3p, hsa-let-7g-5p, hsa-miR-22-3p, hsa-miR-106a-5p, hsa-miR-142-3p, and were associated with OA-related tissues and pathways. Notably, two common targets were identified: Adenosine Triphosphate Citrate Lyase (ACLY), a key enzyme linking citrate metabolism to epigenetic regulation, and phosphoinositide-3-kinase regulatory subunit 1 (PIK3R1), a component of the phosphatidylinositol-3-kinase PI3K/AKT/mTOR pathway. In men, increased miRNA expression may repress ACLY and PIK3R1, affecting catabolic gene expression, inflammation, and OA progression. Conversely, their lower expression in women may mitigate these effects by counterbalancing the OA-promoting influences driven by sex hormones. A functional validation is needed to confirm miRNA–ACLY/PIK3R1 interactions and their sex-specific roles in early OA pathophysiology.

## 1. Introduction

Osteoarthritis (OA) is a prevalent musculoskeletal condition that results in considerable morbidity due to the presence of joint pain and stiffness [1,2]. The etiology of the condition is multifactorial, involving, among others, genetic, biomechanical, and environmental factors [2]. The disease is characterized by the destruction of articular cartilage, together with pathological changes in subchondral bone and associated synovitis [3,4,5].

Within the multifactorial nature of the disease, there is an increasing interest in the dysregulation at a molecular level of the pathogenic processes [6,7,8]. A comprehensive understanding of the molecular mechanisms involved in the pathogenesis of OA is key to the development of personalized therapeutic interventions. Early-stage disease detection is a pivotal challenge in developing therapies for OA. The predominant approach to disease diagnosis and severity assessment is radiographic, though this method is limited to the identification of advanced disease stages and exhibits deficiencies in disease progression monitoring [9,10,11]. In this light, the early identification of non-invasive and sensitive serum/plasma biomarkers could facilitate OA diagnosis, prognosis, and treatment prior to the manifestation of radiographic findings [12,13].

MicroRNAs (miRNAs) are a class of small ribonucleic acids that possess the ability to modify or silence the target sequences of messenger ribonucleic acids (mRNAs), thereby altering the relative expression of genes and the molecular signals that they produce [14]. Their aberrant expression has been reported in a plethora of studies on a wide range of pathological processes [15,16]. With regard to OA, a considerable number of miRNA profiling studies have been conducted on various tissues, resulting in the identification of multiple microRNAs differentially expressed between OA and control joint tissues [17,18]. Nevertheless, the most recent area of investigation is distinguished by the ability to identify miRNAs in liquid biopsies, such as plasma or synovial fluid [19,20,21]. The role of microRNA-203a-3p has been studied both in the serum and synovial fluid of OA patients, showing its involvement in phenotype changes in osteoblasts and synoviocytes [22,23], and in the modulation of the inflammatory response and epithelial-to-mesenchymal transition during OA progression [22]. These findings also corroborated the evidence regarding the possibility that circulating miRNAs could be transferred in body fluids through micro and macro vesicles, exosome, argonaute (Ago) protein complexes, or high-density lipoprotein (HDL). Accordingly, it could serve as a biomarker of the pathophysiological state of the cell and tissue from which the miRNAs are released [21], possibly reflecting the disease evolution [24,25]. In this regard, OA exhibits notable sex-related differences in incidence, pattern of joint involvement, clinical expression, and progression [26,27]. Among the factors influencing these differences, our recent research identified sex-related disparities in the spontaneous osteoclastogenesis of peripheral blood mononuclear cells (PBMCs) derived from patients affected by knee OA [28].

Building upon these observations, the aim of the present study was to preliminarily investigate, through an integrated approach combining in vitro analyses and in silico bioinformatic assessments, the potential of selectively expressed miRNAs as early biomarkers of OA, with a particular focus on their sex-specific expression patterns.

## 2. Results

### 2.1. Patients and Controls

The demographic characteristics of the 40 OA patients (20 women and 20 men) are presented in Table 1. No statistically significant differences were observed between men and women regarding age (*p* = 0.123) or KL grade distribution (*p* = 0.530), whereas BMI showed a significant difference between groups (*p* = 0.011).

### 2.2. Differential miRNAs Expression Profiling and Gene Expression Analysis

**Figure 1:**
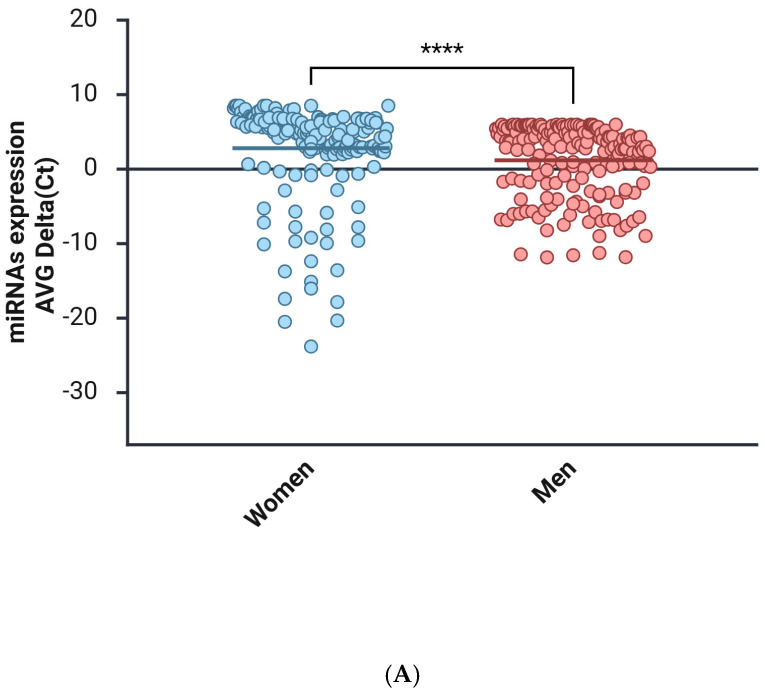

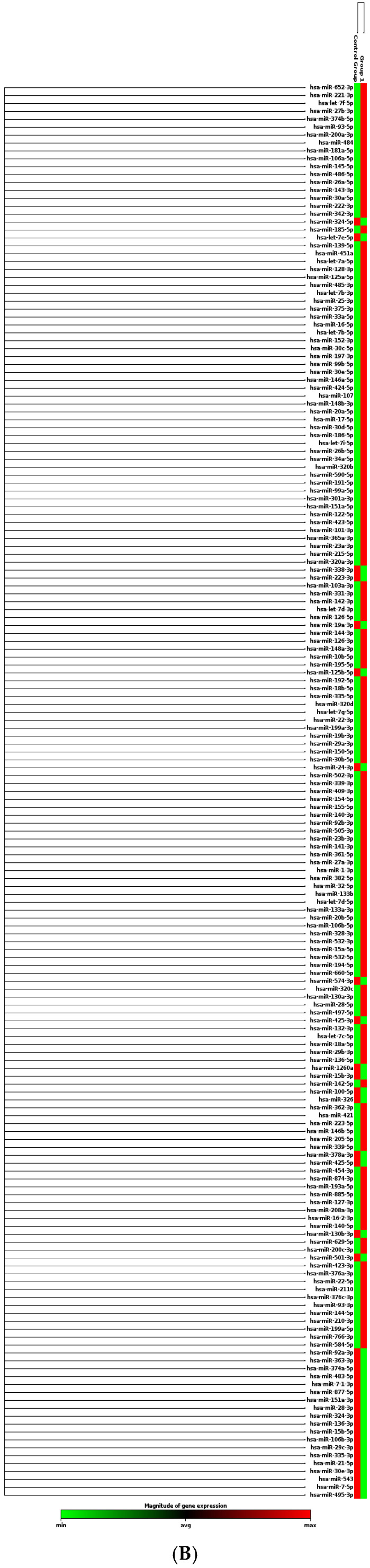
miRNAs expression analysis. (**A**) Distribution of relative miRNAs expression in groups of women and men is represented on a mean/median line graph. Data are analyzed using the Mann–Whitney test (****, *p* < 0.0001). (**B**) Clustergram of miRNAs expression comparing the samples isolated from female (control group) and male (group 1) groups of patients. Clustergrams were built using the miRNAs to show changes between plasma samples derived from patients. The *x*-axis represents all groups listed and the *y*-axis depicts miRNAs expression profiles from all tested samples, normalized on median value of six housekeeping genes. Each colored band represents the expression of a single miRNA from one group: higher expression in red and lower expression in green. Fold-Change (2^^−ΔΔCT^) is the normalized miRNA expression (2^^−ΔCT^) in group 1 divided by the normalized miRNA expression (2^^−ΔCT^) in the control group.

The men and women cohorts were investigated to identify the miRNAs that were altered, comparing the expression in each sex, using the mean Ct value and the NormFinder algorithm (Figure 1). The miRNA expression profiles of the plasma in women were compared with those of their sex counterparts and are summarized in Figure 1A. The miRNAs data are represented as a Clustergram in Figure 1B. To distinguish aberrantly expressed miRNAs from those with equal expression, we selected only miRNAs that were expressed at levels higher than 2-fold or lower than 2-fold (with up- or down-regulation of more than/less than 2 and −2, respectively) and a *p*-value < 0.05.

**Figure 2:**
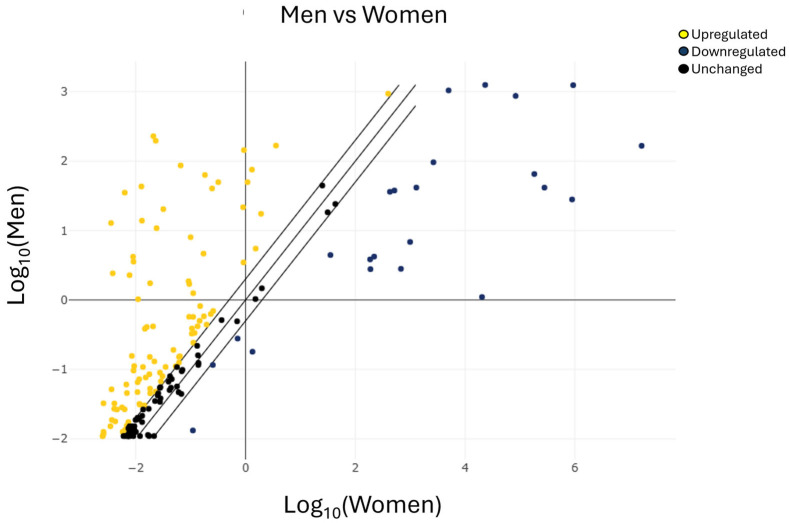
Scatter plot analysis of miRNAs expression in men vs. women. The center diagonal line indicates unchanged miRNA expression, while the outer diagonal lines indicate the selected fold regulation threshold. miRNAs with data points beyond the outer lines in the upper left and lower right corners are upregulated or downregulated, respectively, by more than the fold regulation threshold in the *y*-axis, or the group of male patients, relative to the *x*-axis, or the group of female patients (yellow miRNAs upregulated, blue miRNAs downregulated, and black miRNAs unchanged).

To rapidly visualize large differences in miRNA expression between the two selected groups (Figure 2), a scatter plot analysis was performed by plotting the expression values of one group against those of the other.

### 2.3. Overview of Differently Expressed miRNAs: miRNAs Over-Expressed in Men vs. Women

To identify the miRNAs potentially involved in OA, the list of miRNAs found to be upregulated in men compared to women (although not all reached statistical significance) was used as the input query for the miRNet 2.0 bioinformatics platform. Enrichment analysis performed through the Hypergeometric Test using the “miRNAs–Disease” database revealed that several of these miRNAs are associated with osteosarcoma (6/175 miRNAs; *p* = 0.0135) and osteoarthritis (13/123; *p* = 0.00172) (Table 2).

The analysis of the miRNAs over-expressed in men compared with women using the miRNAs–Tissue database revealed their distribution across several tissues. Notably, significant enrichment was observed in bone marrow (*p* = 0.000076), cartilage (*p* = 0.0302), and bone tissue (*p* = 0.0423) (Table 3). Although peripheral blood and exosomes also showed enrichment for some of the identified miRNAs, these associations did not reach statistical significance.

### 2.4. Overview of Differently Expressed miRNAs: miRNAs Under-Expressed in Men vs. Women

**Figure 3:**
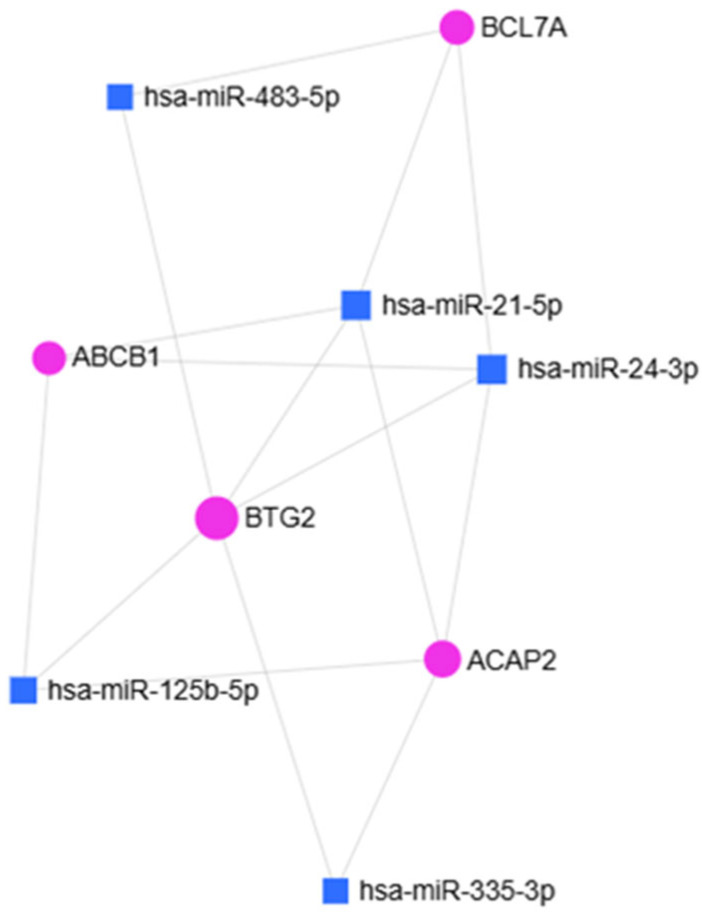
miRNet 2.0 hypergeometric analysis of miRNAs identified through the “miRNAs Disease” database. Squares indicate the miRNAs involved in OA (*p* = 0.0075).

To broadly assess the involvement of miRNAs identified as downregulated in the scatter plot analysis in OA, a bioinformatic analysis was performed using the miRNet platform. Enrichment analysis based on the miRNA–Disease database revealed an association with OA for five miRNAs (*p* = 0.0075): hsa-miR-21-5p, hsa-miR-24-3p, hsa-miR-125b-5p, hsa-miR-335-3p, and hsa-miR-483-5p (Figure 3).

Analysis through the Tissue database revealed that the OA-associated miRNAs were distributed across several tissues. Notably, a significant tissue-specific distribution was observed in bone marrow (*p* = 0.0325), bone (*p* = 0.0207), and cartilage (*p* = 0.0084). In contrast, their distribution in peripheral blood and exosomes did not reach statistical significance (Table 4).

### 2.5. Evaluation of miRNAs Significantly Modulated in Sex-Dependent Manner

**Figure 4:**
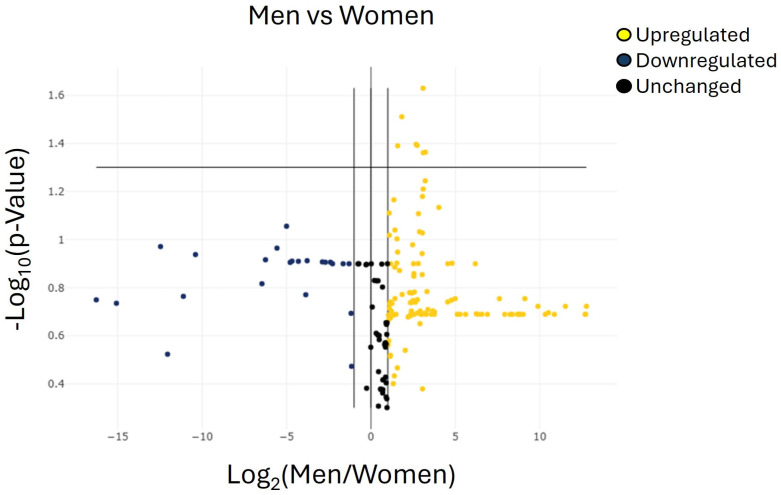
Volcano plots representing the miRNAs with at least 2-fold regulation of expression levels in the group of men group compared with the group of women. The volcano plot identifies significant miRNA expression changes by plotting log 2 of the fold changes in miRNA expression on the *x*-axis versus their statistical significance on the *y*-axis. The center vertical line indicates unchanged miRNA expression, while the two outer vertical lines indicate the selected fold regulation threshold. The horizontal line indicates the selected *p*-value threshold. miRNAs with data points in the far upper left (downregulated) and far upper right (upregulated) sections meet the selected fold regulation and *p*-value thresholds. By combining the fold change results with the *p*-value statistical test results, miRNA with both large and small expression changes that are statistically significant are easily visualized. Table 5 shows the list of miRNAs identified as cut-off criteria (|fold| > 2, *p* < 0.05), represented as dots in the upper right of the graph.

This section summarizes the microRNAs that were differentially expressed with statistical significance in both female and male patients. The expression profiling of the 179 miRNAs described above enabled the identification of sex-associated differences in a cohort of 20 women and 20 men. The volcano plot indicated an overall comparable expression pattern between the two groups, with a cluster of miRNAs showing higher plasma levels in men (Figure 4).

According to the predefined selection criteria (ΔCt ≥ 35 and adjusted *p* < 0.05), seven miRNAs were significantly over-regulated in men compared with women: hsa-miR-106a-5p, hsa-miR-107, hsa-miR-23a-3p, hsa-miR-103a-3p, hsa-miR-22-3p, hsa-miR-142-3p, and hsa-let-7g-5p (Figure 4 and Table 5). No additional miRNAs exhibited statistically significant sex-related differences. Overall, these seven miRNAs emerged as the only candidates displaying robust differential expression between male and female patients in this cohort.

**Figure 5:**
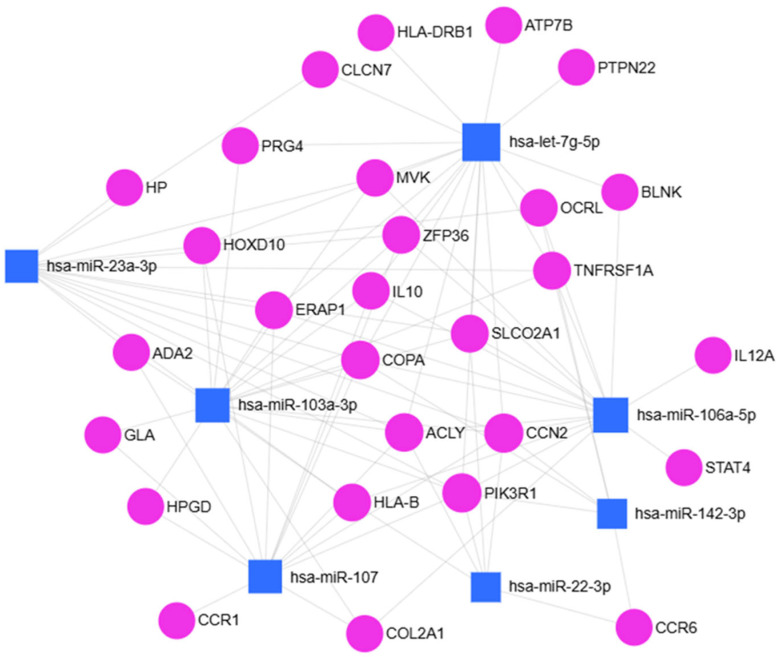
miRNet 2.0 bioinformatic analysis of seven over-regulated miRNAs in men vs. women.

To elucidate the potential biological significance of the over-expressed miRNAs, an in silico analysis was conducted to predict their putative target genes. Two publicly available bioinformatic platforms, miRNet 2.0 and Enrich, together with STRING software, were employed for this purpose. As illustrated in Figure 5, the integrated use of these tools allowed for the identification of a set of predicted target genes for the analyzed miRNAs.

Using the miRNA Disease Database, we obtained a selection of bone disorders in which these miRNAs are implicated. Of particular interest were the following conditions: Arthritis (*p* = 0.0264), Juvenile Arthritis (*p* = 3.46 × 10^−7^), Degenerative Polyarthritis (*p* = 0.00019), and Rheumatoid Arthritis (*p* = 0.00019). Focusing on Arthritis, the genes identified as associated with this disorder were subjected to enrichment analysis using DisGeNET and GeDiPNet_2023 gene sets. These analyses confirmed their involvement in OA, as well as in other related disorders (Figure S1A,B). Further functional classification using Reactome pathways (2024 dataset) revealed their participation in key biological processes, including collagen biosynthesis and modifying enzymes, apoptosis signaling, cell division and viability, syndecan and extracellular matrix (ECM) interactions, and lipid and lipoprotein metabolism (*p* = 0.0328) (Figure S1C).

To elucidate the functional roles and molecular mechanisms of the identified miRNA target genes, Gene Ontology (GO) and Kyoto Encyclopedia of Genes and Genomes (KEGG) pathway analyses were performed using miRNet 2.0 and Enrich. The analysis using the WikiPathways 2024 Human database indicated that several of these target genes are involved in ECM and membrane receptor interactions, Collagen Type III Alpha 1 Chain (COL3A1), Tenascin-XB (TNXB), Collagen Type I Alpha 2 Chain (COL1A2), Collagen Type V Alpha 1 Chain (COL5A1), Collagen Type VI Alpha 2 Chain (COL6A2), Collagen Type V Alpha 2 Chain (COL5A2) and Focal Adhesion/PI3K-Akt-mTOR signaling, Cartilage Oligomeric Matrix Protein (COMP), Collagen Type III Alpha 1 Chain (COL3A1), COL1A2, TNXB, COL5A1, COL6A2, Collagen Type XI Alpha 2 Chain (COL11A2), and Solute Carrier Family 2 Member 1 (SLC2A1) (Figure S2A). Furthermore, the interrogation of the MSigDB Hallmark 2020 dataset revealed enrichment in pathways related to epithelial–mesenchymal transition, oxidative phosphorylation, mechanistic target of rapamycin complex 1 (mTORC1) signaling, hypoxia, androgen response, and PI3K/AKT/mTOR signaling (Figure S2B). The bioinformatic BioPlex2019 analysis highlighted interactions between these target genes and proteins previously implicated in OA, including metalloproteinases 9 and 2 (MMP-9 and MMP-2) (Figure S2C).

### 2.6. Analysis of Common miRNA Target Genes

**Figure 6:**
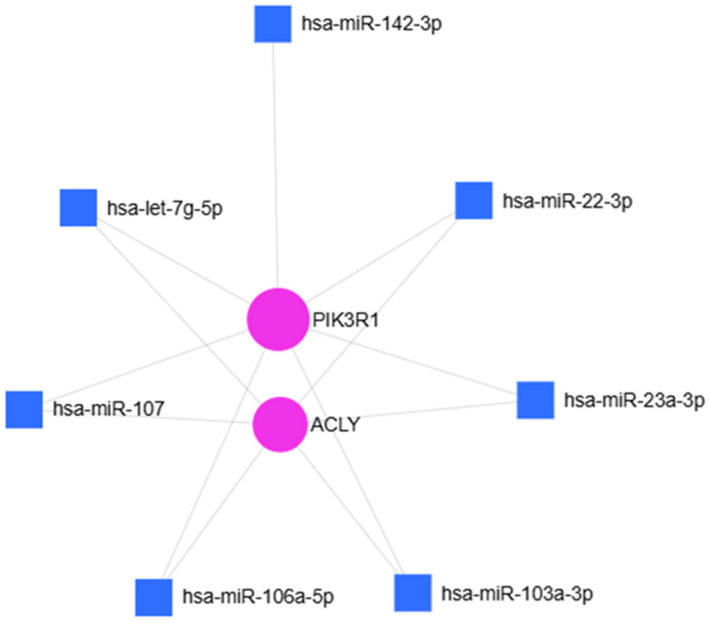
miRNet 2.0 analysis of seven over-regulated miRNAs and their common target-genes. Hypergeometric test and miRNAs “Disease database” used to identify the common targets: ACLY and PI3KR1.

To investigate the potential biological significance of the hsa-miR-107, hsa-miR-23a-3p, hsa-miR-103a-3p, hsa-let-7g-5p, hsa-miR-22-3p, hsa-miR-106a-5p and hsa-miR-142-3p over-regulations in OA disease, an in silico approach to identify their common potential target gene was used. miRNet 2.0 analysis revealed: Adenosine triphosphate citrate lyase (ACLY) and phosphoinositide-3-kinase regulatory subunit 1 (PIK3R1) (Figure 6) as shared targets of these identified miRNAs. ACLY is a key enzyme that generates acetyl-CoA in the nucleocytosolic compartment from mitochondrial-derived citrate for fatty acid and cholesterol biosynthesis as well as histone acetylation [29].

**Figure 7:**
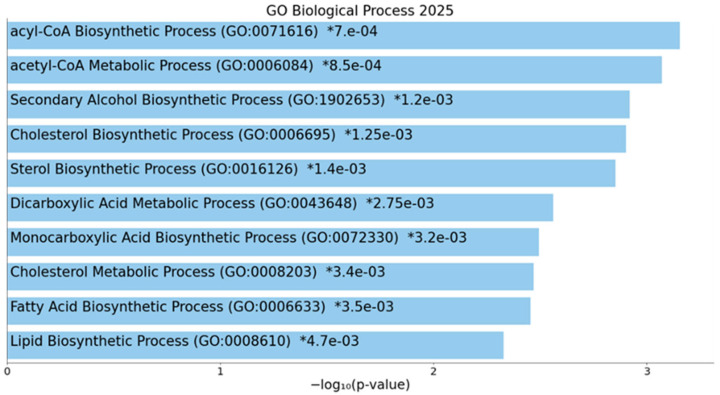
Enrich analysis of ACLY gene. Bar chart of top enriched terms from GO_Biological_Process_2025 gene set library. The top 10 enriched terms for the input gene set are displayed based on the −log10(*p*-value). Coloured bars correspond to terms with significant *p*-values (<0.05). An asterisk (*) next to a *p*-value indicates the term also has a significant adjusted *p*-value (<0.05). The term at the top has the most significant overlap with the input query gene set.

To evaluate the functional role and the molecular mechanisms in which ACLY is involved, miRNet 2.0 and Enrich analyses were performed. The GO: biological process gene data set analysis revealed that ACLY is strongly involved in the acyl-CoA biosynthetic process (GO:0071616) *p* = 0.00070, acetyl-CoA metabolic process (GO:0006084), *p* = 0.00085, cholesterol biosynthetic process (GO:0006695) *p* = 0.00125, etc. (Figure 7).

**Figure 8:**
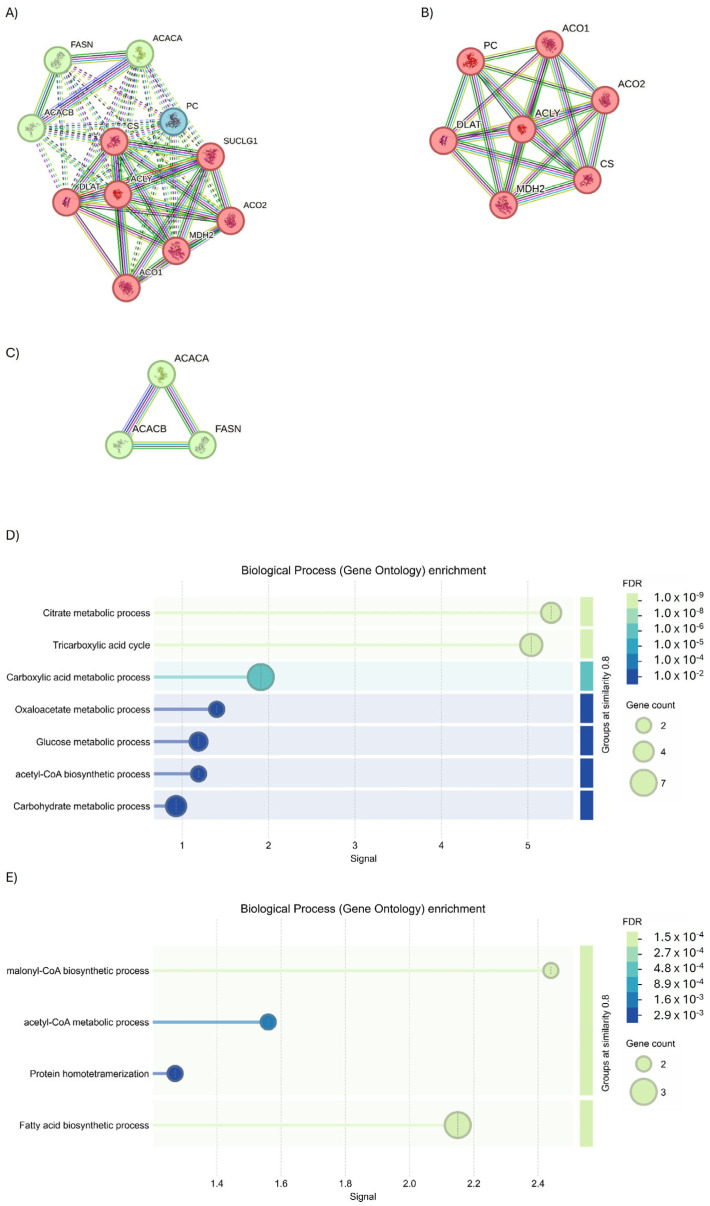
Investigation into the interactors of ACLY using the full STRING network. (**A**) Full string analysis of ACLY. (**B**) Cluster 1 and (**C**) Cluster 2. Node colors indicate the query proteins and first shell of interactors. The edges indicate both functional and physical protein associations (Edge Confidence: medium 0.400 or high 0.700). (**D**) Molecular functions of GO enrichment of Cluster 1 and (**E**) Cluster 2.

**Figure 9:**
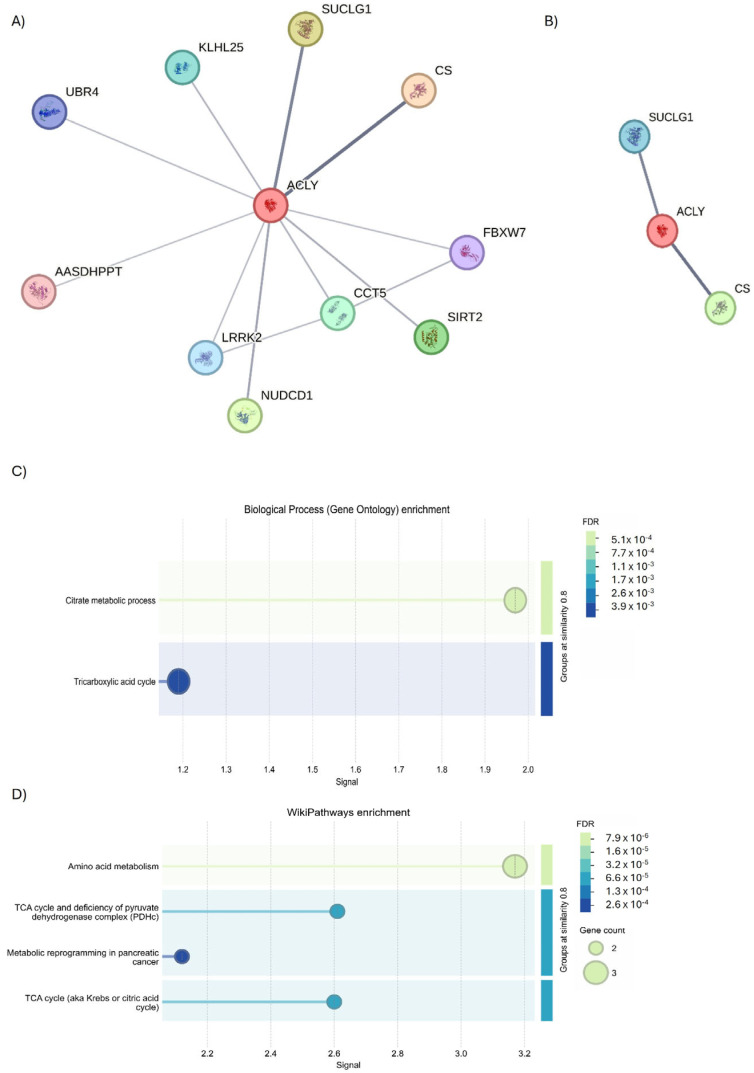
STRING analysis of ACLY. (**A**) Investigation into the interactors of ACLY using the physical subnetwork of STRING. (**B**) Interactors predicted and validated for ACLY. Network nodes represent proteins interactions. Node colors indicate the query proteins and first shell of interactors. The edges indicate that the proteins are part of a physical complex (Edge Confidence: medium 0.400 or high 0.700). (**C**) GO biological process enrichment and (**D**) WiKi Pathways enrichment.

To understand if these common targets may be useful to monitor the evolution of disease, protein–protein interaction (PPI) analysis was performed. Starting from ACLY, the full STRING revealed the presence of two interesting protein clusters (Figure 8A, PPI enrichment *p*-value < 1.0 × 10^−16^ for Cluster 1, and *p* = 0.000449 for Cluster 2). Cluster 1 identified seven genes of interaction implicated in the tricarboxylic acid (TCA) cycle process (Figure 8B), while Cluster 2 identified three genes as interactors involved in fatty acid biosynthesis (Figure 9C).

Following this overview of interactors, an evaluation was conducted using STRING analysis to highlight those that physically interact with the ACLY. The analysis revealed the presence of 11 interactors (Figure 9A); but using an interaction score of 0.700 to define the interaction, a severe physical link between ACLY, citrate synthase (CS), and succinate-CoA ligase alpha (SUCLG1) proteins was observed (Figure 9B). The subsequent biological process analysis demonstrated the function of ACLY and CS (GO: 0046912; *p* = 0.0241) in acyl-CoA ACLY- and CS-mediated acyltransferase activity, whereby acyl groups are converted into alkyl groups during transfer through ACLY and CS (GO: 0046912; *p* = 0.0241) (Figure 9C). Moreover, the Wiki Pathways analysis indicated the participation of ACLY, CS, and SUCLG1 (hsa00020; *p* = 0.00019) in the TCA cycle pathway (Figure 9D).

**Figure 10:**
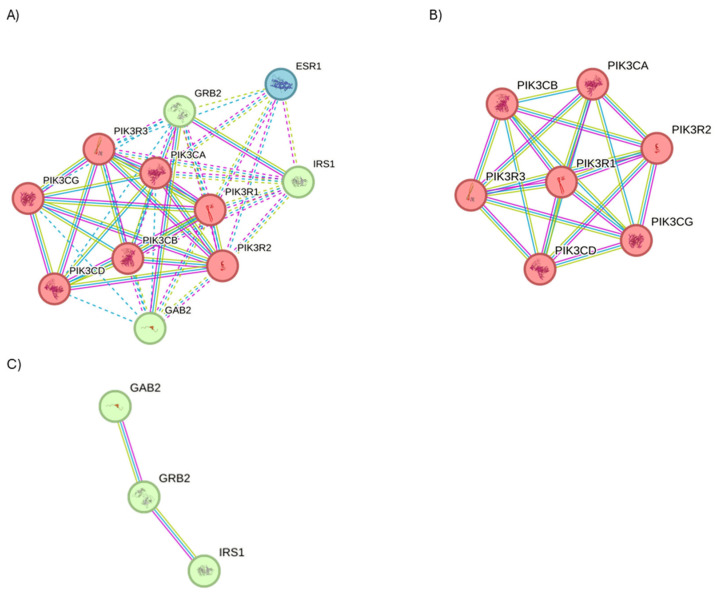
STRING analysis of PIK3R1. (**A**) Investigation on the interactors of PIK3R1 using the physical subnetwork of STRING. (**B**) Cluster 1 and (**C**) Cluster 2. Interactors predicted and validated of PIK3R1. Network nodes represent protein interactors. Node colors indicate the query proteins and the first shell of interactors. The edges indicate that the proteins are part of a physical complex (Edge Confidence: medium 0.400 or high 0.700).

STRING analysis was performed to examine the PPI network applying the full STRING network filter (*p*-value: 7.92 × 10^−12^) on PIK3R1. Three interesting protein clusters (Figure 10A, PPI enrichment *p*-value < 1.0 × 10^−16^ for both clusters; *p* = 0.000449 for Cluster 2) were identified. Cluster 1 is composed of seven genes, identified the fhosphatidylinositol 3-kinase complex, class IA (Figure 10B); Cluster 2 is characterized by three genes involved in transmembrane receptor protein tyrosine kinase adaptor activity, in PI3K cascade and in erythropoietin signaling, and the insulin receptor complex (Figure 10C); while Cluster 3 is composed of 1 gene, involved in estrogen receptor 1 (ESR1) signaling.

**Figure 11:**
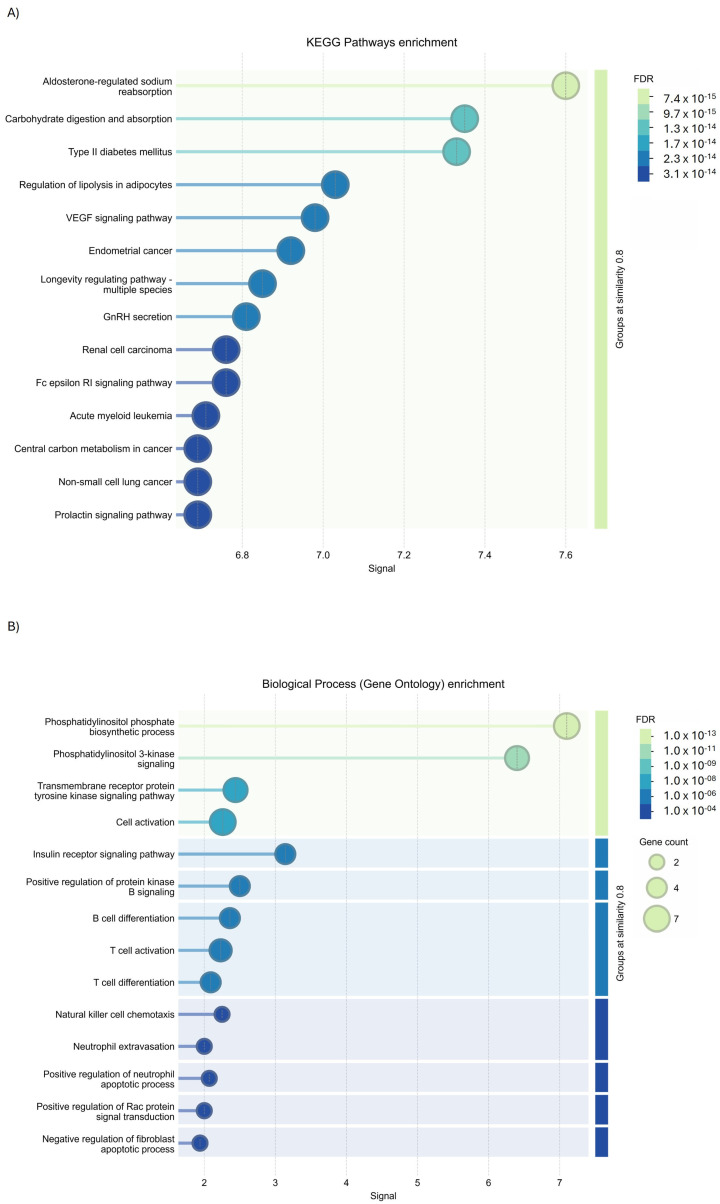

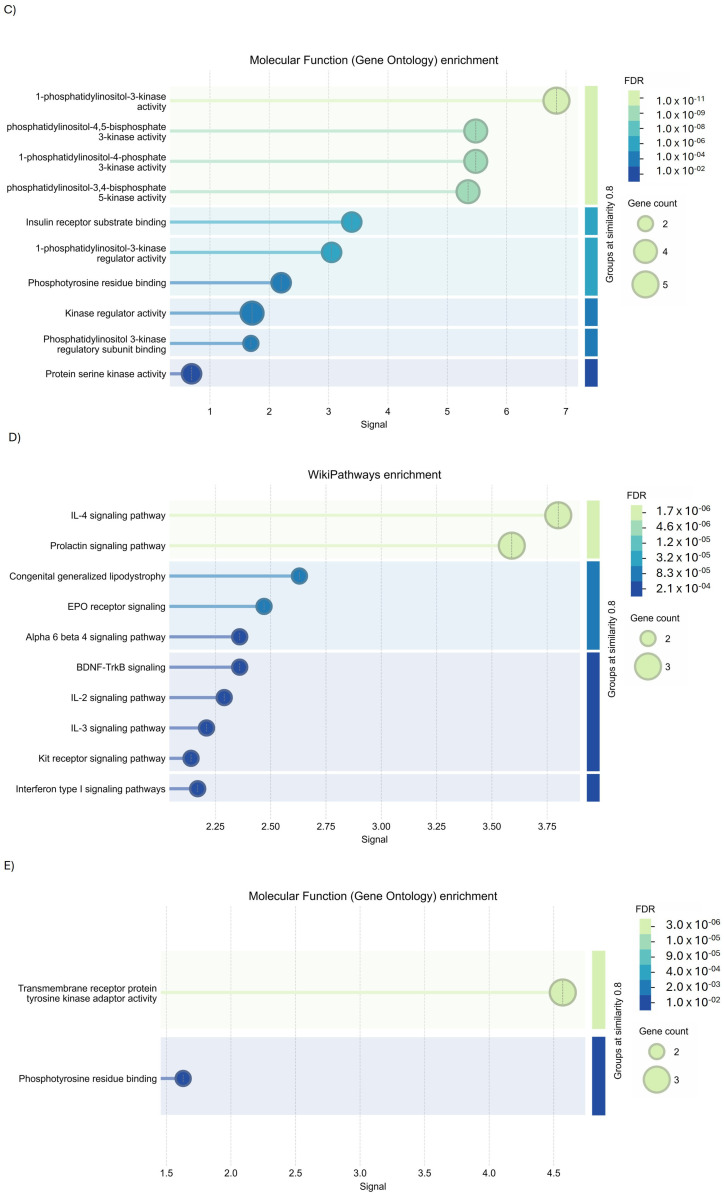
GO analysis of PIK3R1 using STRING tools. (**A**) KEGG Pathways enrichment, (**B**) biological process enrichment, (**C**) molecular function enrichment of Cluster 1, (**D**) Wiki Pathways enrichment, and (**E**) molecular function enrichment of Cluster 2.

GO analysis using KEGG Pathways enrichment of Cluster 1 identified their involvement (PPI enrichment *p*-value: <1.0 × 10^−16^) in the regulation of lipolysis in adipocytes, the vascular endothelial growth factor (VEGF) signaling pathway, and aldosterone-regulated sodium reabsorption carbohydrate digestion and absorption (Figure 11A); while molecular function (Figure 11B) and biological process enrichment revealed Cluster 1 to be involved in different inflammatory pathways (Figure 11C). Concerning the analysis of Cluster 2, GO investigation via the Wiki database (Figure 11D) and molecular function enrichment (Figure 11E) underlined their role in interleukin 2 (IL-2) signaling and tyrosine kinases signaling.

## 3. Discussion

The main aim of this study is to evaluate disease progression and determine the most appropriate medical intervention with a sex-specific focus, which is essential to improve our understanding of the manifestations of OA in male and female patients. In this regard, the expression of miRNAs has recently been the focus of considerable attention, primarily due to their multifaceted impact on downstream signaling pathways, including those associated with immune-related cytokines. As demonstrated in our recent study [28], PBMCs isolated from OA patients exhibited a higher propensity for spontaneous osteoclastogenesis, particularly in male patients, when compared to the female patients. To understand the reasons relating to this phenomenon, data regarding plasma miRNAs was collected to preliminarily investigate a possible candidate circulating miRNA associated with sex in early OA disease. Our in silico analysis showed that several miRNAs were modulated, although not in a significant manner, while hsa-miR-107, hsa-miR-23a-3p, hsa-miR-103a-3p, hsa-let-7g-5p, hsa-miR-22-3p, has-miR-106a-5p, and miR-142-3p were differently expressed in men compared to women in a statistically significant manner. The miRNet 2.0 analysis through the miRNA “Disease” database indicated that these miRNAs are involved in OA and expressed, as established using the miRNA Tissue database, on cartilage, bone, and peripherical blood. In the literature only limited evidence is available about the identification of these miRNAs in liquid biopsy derived from OA patients. miR-23a-3p has been previously reported as differentially expressed in the plasma of OA patients [30], where its circulating levels were associated with disease-related clinical features, supporting its potential role as a circulating biomarker in OA. In contrast, for the remaining miRNAs identified, including miR-107 [31], miR-103a-3p, let-7g-5p, miR-22-3p, miR-106a-5p, and miR-142-3p [32,33], no studies have so far specifically documented their differential expression in serum or plasma samples from OA patients. Nevertheless, several of these miRNAs have been implicated in OA-related biological processes at the tissue or cellular level, such as cartilage homeostasis, inflammatory signaling, and metabolic regulation, suggesting a potential pathophysiological relevance.

Concerning this, GO-Reactome and Wiki Pathways analysis revealed the involvement of these miRNAs in the regulation of collagen biosynthesis and modifying enzymes, apoptosis signaling, cell division and viability, syndecan and ECM interactions, and metabolism of lipids and lipoproteins. Finally, MSigDB Hallmark 2020 data set analysis underlined their involvement in epithelial–mesenchymal transition, oxidative phosphorylation, mTORC1 signaling, hypoxia, androgen response, and PIK/AKT/mTOR; and using BioPlex2019, the interactions of these genes with some proteins involved in OA, such as MMP-9 and MMP-2, were shown.

The lack of clinical evidence in liquid biopsies highlights the exploratory nature of the present study and underscores its novelty, as it provides preliminary data on circulating miRNAs that have not yet been extensively investigated in OA patient cohorts.

Following the aim to identify possible miRNAs involved in OA by using a liquid biopsy and the subsequent hypothesis to find the common gene targets regulated by them and suitable for OA diagnosis and therapy, in silico analysis was performed. miRNet 2.0 analysis predicted that hsa-miR-107, hsa-miR-23a-3p, hsa-miR-103a-3p, hsa-let-7g-5p, hsa-miR-22-3p, has-miR-106a-5p, and miR-142-3p have binding sequences complementary to the 3′ untranslated region (3′UTR) of ACLY and to 3′UTR of PIK3R1. This is an interesting finding, suggesting that these miRNAs may contribute to modulating ACLY and PIK3R1 expressions under OA conditions. ACLY is a key metabolic enzyme involved in the generation of acetyl-CoA and the regulation of lipid metabolism and epigenetic modifications, and it plays an important role linking glucose metabolism to chromatin modification and global transcription [34,35]. To our knowledge, this is the first report that suggests a potential post-transcriptional regulatory axis between miRNAs and ACLY in the context of OA.

ACLY is identified as a critical player in the pathogenesis of OA, the functionality of chondrocytes, and cartilage degradation. ACLY is upregulated in OA chondrocytes and promotes the generation of acetyl-CoA, which in turn contributes to histone acetylation and the transcription of pro-catabolic and inflammatory genes, including MMP13 and inducible nitric oxide synthase (iNOS) [36]. Also, OA chondrocytes undergo a metabolic reprogramming in which ACLY-derived acetyl-CoA fuels epigenetic modifications that promote cartilage matrix degradation. Thus, ACLY functions as a metabolic–epigenetic bridge linking altered glucose/citrate flux to the transcriptional dysregulation in chondrocytes and the degradation of OA cartilage. ACLY has also been associated with NF-κB signaling, and its inhibition has been shown to attenuate inflammation, reduce matrix degradation, and enhance the expression of anabolic markers such as collagen 2a 1 (COL2A1) and aggrecan (ACAN) [33]. Altered ACLY activity is observed in metabolic disorders, and several metabolic syndromes, such as dyslipidemia, obesity, and insulin resistance, have been linked to OA [34,37].

The STRING analysis of ACLY interactors reported the interaction between ACLY, CS, and SUCLG1 and the GO analysis underlining their involvement in citrate metabolism and acyltransferase activity. CS catalysis is the first irreversible step of the TCA cycle, condensing acetyl-CoA with oxaloacetate to form citrate, and it is essential for mitochondrial oxidative metabolism. Regarding the role of CS in OA, in the literature it has been demonstrated that in human OA chondrocytes mitochondrial respiratory activity is altered, and the upregulation of TCA cycle enzymes in senescent or hypertrophic OA chondrocytes has been observed [38]; finally, it was demonstrated that serum citrate concentrations, which reflect CS/TCA activity, are inversely correlated with OA severity [39], MMP-13 levels, and structural joint changes [39].Therefore, CS alterations may indicate mitochondrial dysfunction and a metabolic shift in OA chondrocytes from oxidative phosphorylation to catabolic/inflammatory metabolism, contributing to disease progression. Meanwhile, SUCLG1, which encodes to the α-subunit of succinate-CoA ligase (ADP/GDP-forming), catalyzes the reversible conversion of succinyl-CoA to succinate and ATP/GTP in the TCA cycle, the only substrate-level phosphorylation step within the cycle. No evidence has been obtained until now on its role in OA, but the reduction in SUCLG1 expression has been demonstrated in aging and cancer, conditions characterized by mitochondrial dysfunction and altered energy metabolism. Nevertheless, given its role in the TCA cycle, impaired SUCLG1 activity could hypothetically contribute to mitochondrial energy deficits, succinyl-CoA accumulation, and redox imbalance in OA chondrocytes. SUCLG1 may participate in OA-related mitochondrial dysfunction, but currently our evidence is minimal. Overall, this remains speculative and requires direct investigation. Subsequent studies will be conducted to evaluate the variations in plasma citrate levels observed in OA patients and to determine their prospective function as direct or indirect biomarkers of disease progression in correlation with sex.

Regarding the link between the seven over-regulated miRNAs in men’s vs. women’ plasma and PIK3R1 mRNA regulation, the role of PIK3R1 and its relative signaling PI3K/AKT/mTOR pathway is known in OA progression [40]. In fact, the gene PIK3R1 encodes to the regulatory subunit p85α of the class IA phosphoinositide-3-kinases (PI3Ks) and plays a pivotal role in the PI3K/AKT signaling pathway [41]. The joint OA niche is predominantly characterized by affected cells (chondrocytes and synovial cells) that produce excessive inflammatory cytokines (such as interleukin-1β (IL-1β), transforming growth factor β (TGF-β) and NO) to promote cartilage degradation and synovial inflammation. PIK3R1 has been identified as a critical modulator of cartilage cell fate (proliferation, apoptosis, autophagy), ECM turnover, stress responses, synovial inflammation, subchondral bone sclerosis, and the inflammation state of the OA microenvironment [42,43,44,45]. It has also been demonstrated that PI3K/AKT is involved in the proliferation and migration of fibroblast-like synoviocytes (FLSs), which may contribute to cartilage damage, and involved in the inflammatory response of cytokines, also activating the mTOR pathway [46]. This is also a key metabolic signaling pathway involved in subchondral bone changes during OA progression. Recent studies have indicated that the dysregulation of PIK3R1 may potentially contribute to pathological signaling. miR-155 directly binds and inhibits PIK3R1, resulting in reduced P85α expression, improving a decrease in AKT phosphorylation and inducing (i) an increase in caspase-3 cleavage; (ii) a decrease in collagen II and aggrecan expression; and (iii) an increase in MMP3 and MMP13 production in IL-1β-stimulated human OA cells [47]. In addition, an in vivo study using an PI3K/AKT inhibitor treatment demonstrated a reduction in subchondral bone sclerosis activating less osteogenesis in OA mice, instantaneously moderating cartilage erosion [48]. This evidence was also supported by an ex vivo study reporting a downregulation of PI3K/AKT signaling in human OA cartilage tissues vs. healthy [49], confirmed by an in vitro study performed in an OA model on chondrocytes, treated with IL-1β or tumor necrosis factor (TNF-α), in which PI3K/AKT was under-regulated compared to in untreated cells [50]. Based on these observations, the present study provides preliminary evidence suggesting that the miRNAs found to be upregulated in the plasma of male compared with female OA patients may be released from OA-affected tissues into the circulation and potentially contribute to the coordination of disease-related mechanisms by targeting and downregulating ACLY and PIK3R1. This mechanism may thereby contribute to sex-specific differences in OA pathophysiology by modulating metabolic and signaling pathways in a concerted manner and probably explains the differences in spontaneous osteoclastogenesis documented in PBMCs isolated from male and female OA patients [28]. The sex-expression differences are probably derived from the hormonal differences present between the two sexes [49]. Within this conceptual framework, the current findings support our working hypothesis regarding the potential mechanism of action of the identified miRNAs. Specifically, in male patients, the upregulation of hsa-miR-107, hsa-miR-23a-3p, hsa-miR-103a-3p, hsa-let-7g-5p, hsa-miR-22-3p, hsa-miR-106a-5p, and hsa-miR-142-3p may be associated with altered or inhibited expression of ACLY and PIK3R1, thereby contributing to cartilage deterioration, synovial phenotype remodeling, and a pro-inflammatory microenvironment. Conversely, in female patients, the relative downregulation of these miRNAs may represent a compensatory mechanism limiting cartilage and synovial tissue degeneration by counteracting miRNA-mediated gene dysregulation and partially offsetting the pro-osteoarthritic effects of sex-specific hormonal imbalance, lifestyle factors, and aging.

Despite these promising insights, it is important to emphasize that this study is preliminary and observational in nature and therefore has inherent limitations. The interactions between miRNAs and ACLY or PIK3R1 were identified using in silico prediction tools and require experimental validation through approaches such as luciferase reporter assays and the use of miRNA mimics or inhibitors. Moreover, functional studies are needed to determine whether modulation of these miRNAs can influence the OA phenotype both in vitro and in vivo, and whether these effects are specifically mediated through ACLY- or PIK3R1-dependent mechanisms. Importantly, the present findings are based on an initial, limited cohort. Evaluations on a larger patient population, including male and female control/healthy patients and multiple treatment time points and additional clinical and molecular endpoints, are planned as part of the second phase of the ongoing project. The encouraging results obtained in this preliminary study provide a rationale for future validation and correlation studies, which will be carried out in follow-up investigations to confirm the relevance of these miRNAs and their potential sex-specific roles in OA pathophysiology. Finally, the temporal dynamics of PI3K/AKT/mTOR pathway activation at different stages of OA, as well as cell-type-specific responses within the joint microenvironment (including chondrocytes, synoviocytes, and subchondral osteoblasts), remain to be fully characterized. Such analyses will be crucial to assess potential sex-specific temporal expression patterns during OA progression [48,51,52].

## 4. Materials and Methods

### 4.1. Enrolled Patients

Patients were recruited from an ongoing randomized controlled trial (RCT) investigating injectable treatments for knee OA [53], whose inclusion and exclusion criteria are detailed in Table 1. The study received approval from the Ethics Committee on 23 May 2023 (CE AVEC: 150/2023/Sper/IOR), and informed consent was obtained from all participants prior to enrollment. The ongoing RCT includes patients with KL I-IV, with inclusion and exclusion criteria shown in Table 6. In the present study we selected only patients with KL I-II “early OA” (n = 40; 20 women, or control group, and 20 men or group 1). The information regarding comorbid conditions and other clinical data of the enrolled patients will be updated at the end of patient recruitment, in accordance with the guidelines of the Ethics Committee.

### 4.2. Sample Plasma Collection

Whole peripheral blood obtained from human OA patients was collected in sterile BD Vacutainer Venous tubes containing the anticoagulant ethylenediaminetetraacetic acid (EDTA). Samples were centrifuged at 2000× *g* for 15 min in a refrigerated centrifuge. The resulting plasma layers were harvested, aliquoted, and stored at −80 °C. Upon conclusion of sample collection, 200 µL of plasma from each specimen was used for the subsequent analyses.

### 4.3. miRNA Purification and Amplification

Total miRNAs from biological fluids were extracted using the commercially available miRNeasy Serum/Plasma Advanced Kit (217204, Qiagen, Milan, Italy), 200 µL of plasma was processed according to the manufacturer’s instructions, and reverse transcription was performed using a miRCURY LNA RT Kit (Qiagen, Milan, Italy). miRNA expression was evaluated using a miRCURY LNA SYBR Green PCR Kit (339347, Qiagen, Milan, Italy) and the miRCURY LNA™ miRNA Focus Panel–Serum/Plasma (YAHS-106YA Panel I and IIA, 339325, Qiagen, Milan, Italy) following the instructions reported in the datasheets. Real-time PCR was performed using the following cycling conditions: an initial heat activation step at 95 °C for 2 min, followed by 40 cycles of a two-step amplification protocol consisting of denaturation at 95 °C for 10 s and combined annealing/extension at 56 °C for 60 s. The analysis of miRNA expression between the groups of men and women was conducted using the GeneGlobe analysis web through the representation of fold change (2^^−ΔCt^) and bioinformatic evaluation with miRNet 2.0 and Enrichr tools. For the definition of the significant expression of miRNAs identified, a 2-fold modulation between the tested groups and a *p* < 0.05 limit was used (Ct cut-off: 35; *p*-value threshold: 0.05; fold-regulation threshold: 2) and normalization analysis was performed using NormFinder algorithm. This is an algorithm that attempts to find the optimum reference miRNAs out of a group of candidate miRNAs (hsa-miR-451a, hsa-miR-223-3p, hsa-miR-19a-3p) [54].

### 4.4. Bioinformatic Analysis

miRNet software 2.0 analysis was employed to predict the target genes of the significantly upregulated miRNAs. Recovery of the results was obtained using the miRNet database (https://www.mirnet.ca/faces/home.xhtml, last software update 9 August 2025), which permits the integration of 11 existing miRNA target prediction programs (TarBase, miRTarBase, miRecords, miRanda, miR2Disease, HMDD, PhenomiR, SM2miR, PharmacomiR, Epi-miR, and starBase), while the Enrich database was used to investigate the signaling in which miRNAs and their targets are involved [31,55,56].

### 4.5. Protein Interaction Bioinformatic Analysis

Protein–protein interactions were evaluated using the STRING database (Search Tool for the Retrieval of Interacting Genes/Proteins; v11.5 designated by Global Biodata Coalition and ELIXIR), which compiles evidence for both physical and functional associations. In the resulting network, nodes denote proteins, whereas edges represent inferred protein–protein relationships. Protein clustering was performed by applying K-means analysis (K = 3) using the computational tools implemented in STRING.

### 4.6. Statistical Analysis

Apart from the statistical analyses conducted on the data using the bioinformatics software, the clinical data and differences in the distribution of relative miRNA expression in groups of women and men were analyzed using GraphPad Prism software (version 9.0.0, GraphPad Software, San Diego, CA, USA) and applying the non-parametric Mann–Whitney U test. A *p*-value of less than 0.05 was deemed to be statistically significant.

## 5. Conclusions

These findings provide a preliminary foundation for future studies aimed at validating this miRNA–target network and exploring its potential therapeutic relevance, including possible sex-specific effects. From a translational perspective, the identified miRNAs may represent candidate biomarkers for OA progression, while serum or plasma citrate levels could serve as a non-invasive indicator of disease severity and metabolic status. Additionally, CS and SUCLG1 may offer insights into mitochondrial dysfunction in OA. It is important to emphasize that the present results are exploratory and based on a limited cohort; moreover, the study design did not include a matched healthy control group, as this was not foreseen within the protocol approved by the Ministry, which should be considered when interpreting the findings. Future studies will therefore incorporate appropriate male and female control groups to determine whether the molecular signatures reported in this work are associated with early-stage OA or reflect more general disease- or metabolism-related alterations. Ultimately, integrating these preliminary findings within multi-omics frameworks may contribute to the development of personalized therapeutic strategies for OA.

## Figures and Tables

**Table 1:** Patient characteristics. Mean ± SD [95%CI].

Characteristic	Women (n = 20)	Men (n = 20)	*p*-Value
Age (years)	56.0 [52.4–59.5]	58.8 [55.7–61.8]	0.123
BMI (kg/m^2^)	24.3 [22.4–26.1]	26.5 [25.5–27.6]	0.011
KL (grade)	KL I = 9; KL II = 11	KL I = 11; KL II = 9	0.530

BMI = Body mass index; KL = Kellgren–Lawrence; SD = Standard deviation; CI = confidence interval.

**Table 2:** List of miRNAs over-expressed in men vs. women reported to be involved in bone disease. miRNAs defined in the table were obtained through miRNet 2.0 analysis using the miRNA “Disease” database.

Osteosarcoma	Osteoarthritis
hsa-let-7d-5p	hsa-miR-15a-5p
hsa-miR-19b-3p	hsa-miR-23a-3p
hsa-miR-1-3p	hsa-miR-26b-5p
hsa-miR-127-3p	hsa-miR-27a-3p
hsa-miR-155-5p	hsa-miR-199a-5p
hsa-miR-146b-5p	hsa-miR-27b-3p
	hsa-miR-130a-3p
	hsa-miR-127-3p
	hsa-miR-146a-5p
	hsa-miR-365a-3p
	hsa-miR-335-5p
	hsa-miR-146b-5p
	hsa-miR-140-3p

**Table 3:** List of miRNAs over-expressed in men vs. women divided based on tissue-specific expression. The miRNAs reported in the table were obtained through miRNet 2.0 analysis using the miRNA “Tissue” database. Highlighted in yellow, the miRNAs identified as involved in OA following the miRNAs Disease database.

Bone Marrow	Bone	Cartilage
hsa-let-7c-5p	hsa-let-7c-5p	hsa-miR-146a-5p
hsa-let-7d-5p	hsa-let-7d-5p	
hsa-let-7f-5p	hsa-let-7f-5p	
hsa-miR-15a-5p	hsa-miR-23a-3p	
hsa-miR-19b-3p	hsa-miR-335-5p	
hsa-miR-23a-3p		
hsa-miR-26b-5p		
hsa-miR-27a-3p		
hsa-miR-103a-3p		
hsa-miR-199a-5p		
hsa-miR-1-3p		
hsa-miR-27b-3p		
hsa-miR-130a-3p		
hsa-miR-127-3p		
hsa-miR-146a-5p		
hsa-miR-155-5p		
hsa-miR-365a-3p		
hsa-miR-335-5p		
hsa-miR-20b-5p		
hsa-miR-146b-5p		
hsa-miR-140-3p		

**Table 4:** Summary of down-expressed miRNAs in men vs. women in different tissues.

Bone Marrow	Bone	Cartilage
hsa-miR-21-5p	hsa-miR-21-5p	hsa-miR-125b-5p
hsa-miR-24-3p	hsa-miR-483-5p	
hsa-miR-125b-5p		
hsa-miR-335-3p		
hsa-miR-483-5p		

**Table 5:** List of significantly upregulated miRNAs.

miRNA IDs	Fold Regulation	*p*-Value
hsa-miR-106a-5p	3.00	0.040702
hsa-miR-107	8.57	0.043502
hsa-miR-23a-3p	8.49	0.023443
hsa-miR-103a-3p	6.67	0.040528
hsa-miR-142-3p	3.55	0.030819
hsa-let-7g-5p	6.38	0.040143
hsa-miR-22-3p	9.35	0.043272

**Table 6:** Inclusion and exclusion criteria.

Inclusion Criteria	Exclusion Criteria
History of chronic pain or swelling (at least 6 months); Age 40–75 years; KL grade I–IV; No benefit after at least 4 months of nonoperative treatment.	Age < 40 and >75 years; Major coronal deviation > 5°; History of trauma or intra-articular injection therapy within 6 months before treatment; Knee surgery within 12 months; Presence of any concomitant knee lesion causing pain or swelling other than OA; Neoplasm, infections, systemic disorders.

KL = Kellgren–Lawrence; OA = Osteoarthritis.

## Data Availability

The datasets used and/or analyzed during the current study are available from the corresponding author on reasonable request.

## Supplementary Materials

The following supporting information can be downloaded at https://www.mdpi.com/article/10.3390/ijms27021019/s1.

## Abbreviations

The following abbreviations are used in this manuscript:

miRNAs	MicroRNAs
3′-UTR	3′-untranslated regions
OA	Osteoarthritis
PBMC	Peripheral blood mononuclear cells
KL	Kellgren–Lawrence grading
ECM	Extracellular matrix
TNF-α	Tumor necrosis factor a
IL-1β	Interleukin-1β
ACAN	Aggrecan
PI3K-Akt	Phosphoinositide 3-kinase/protein kinase B
PIK3R1	Phosphoinositide-3-kinase regulatory subunit 1
TGF-β	Transforming growth factor, beta
ACLY	ATP-citrate lyase
TNXB	Tenascin-XB
COL1A2	Collagen Type I Alpha 2 Chain
COL5A1	Collagen Type V Alpha 1 Chain
COL6A2	Collagen Type VI Alpha 2 Chain
COL5A2	Collagen Type V Alpha 2 Chain
COMP	Cartilage Oligomeric Matrix Protein
COL3A1	Collagen Type III Alpha 1 Chain
COL11A2	Collagen Type XI Alpha 2 Chain
SLC2A1	Solute Carrier Family 2 Member 1
PPI	Protein–protein interaction
MMP-	Metalloproteinases
Ago	Argonaute
HDL	High-density lipoprotein
CS	Citrate synthase
SUCLG1	succinate-CoA ligase alpha
TCA	tricarboxylic acid cycle
