# Supplementary material for: Investigating Sex-Linked miRNAs for Potential Osteoarthritis Therapy Biomarkers
Source: Int J Mol Sci. 2026 Jan 20;27(2):1019. doi: 10.3390/ijms27021019 (PMC12842146; doi:10.3390/ijms27021019)
Supplement: Supplementary file 1 [file ijms-27-01019-s001.zip › ijms-4053090-supplementary.pdf]

International Journal of *Molecular Sciences*

MDPI

Article

Investigating Sex-Linked miRNAs for Potential Osteoarthritis Therapy Biomarkers

Supplementary Figures

A

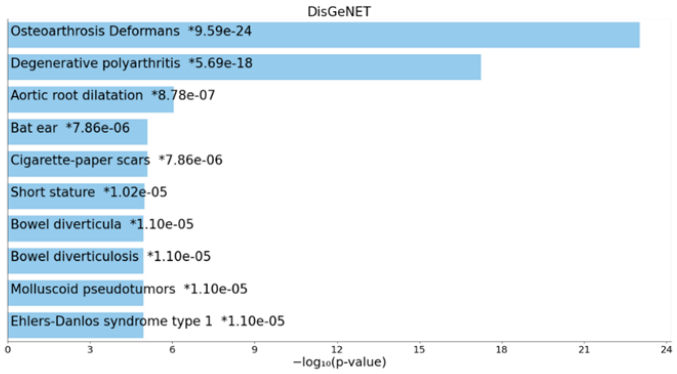

B

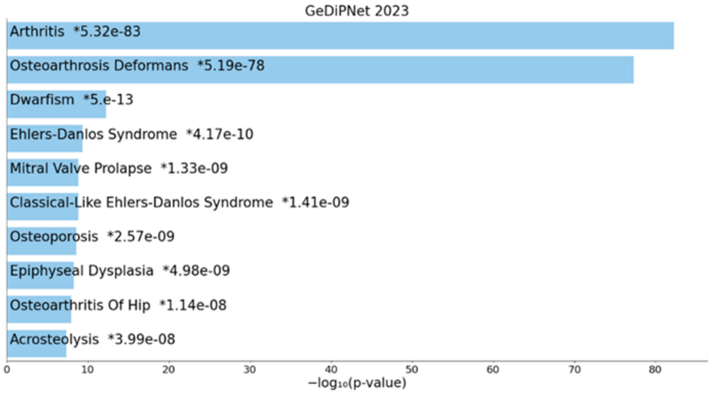

C

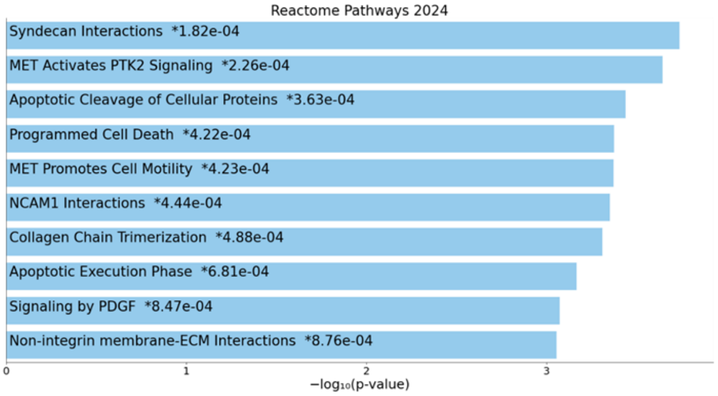

Supplementary Figure S1. Enrich analysis. Bar chart of top enriched terms from the A) DisGeNET, B) GeDiPNet_2023, C) Reactome_Pathways_2024 gene set library. The top 10 enriched terms for the input gene set are displayed based on the -log10(p-value), with the actual p-value shown next to each term. The term at the top has the most significant overlap with the input query gene set.

A

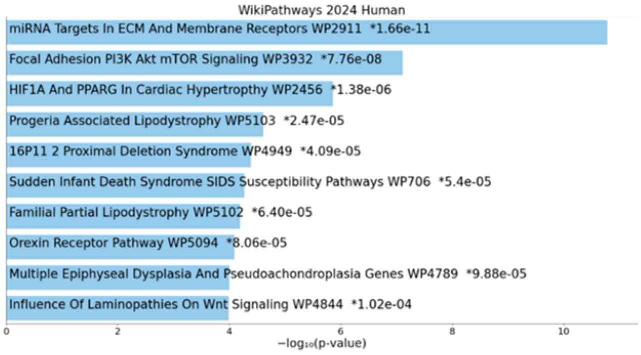

B

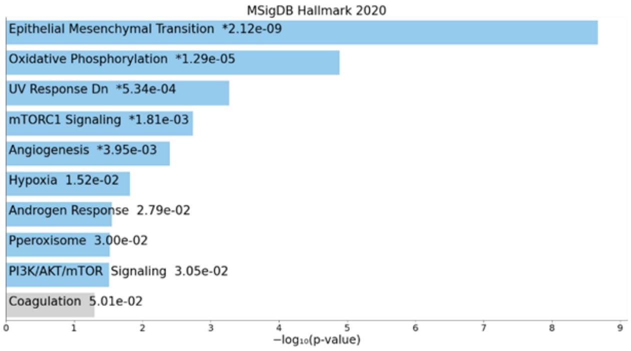

C

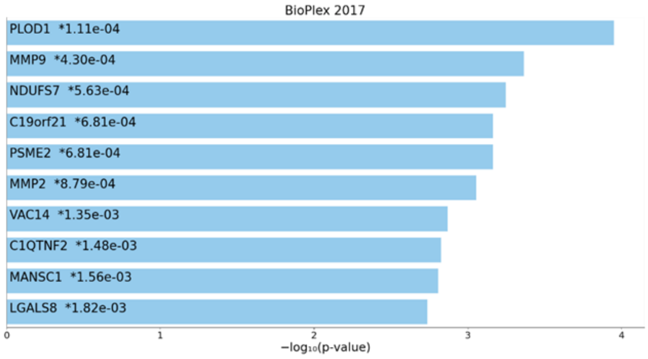

Supplementary Figure S2. Bar chart of top enriched terms from the Enrich software. A) WikiPathways_2024_Human and B) MSigDB hallmarks 2020 and C) BioPlex 2017 gene set. The top 10 enriched terms for the input gene set are displayed based on the -log10(p-value), with the actual p-value shown next to each term. The term at the top has the most significant overlap with the input query gene set.
